# Disrupted resting-state brain functional network in methamphetamine abusers: A brain source space study by EEG

**DOI:** 10.1371/journal.pone.0226249

**Published:** 2019-12-11

**Authors:** Hassan Khajehpour, Bahador Makkiabadi, Hamed Ekhtiari, Sepideh Bakht, Alireza Noroozi, Fahimeh Mohagheghian

**Affiliations:** 1 Department of Medical Physics and Biomedical Engineering, School of Medicine, Tehran University of Medical Sciences (TUMS), Tehran, Iran; 2 Research Center for Biomedical Technology and Robotics (RCBTR), Institute of Advanced Medical Technologies (IAMT), Tehran University of Medical Sciences (TUMS), Tehran, Iran; 3 Laureate Institute for Brain Research (LIBR), Tulsa, OK, United States of America; 4 Iranian National Center for Addiction Studies (INCAS), Tehran University of Medical Sciences (TUMS), Tehran, Iran; 5 Department of Cognitive Psychology, Institute for Cognitive Sciences Studies (ICSS), Tehran, Iran; 6 Neuroscience and Addiction Studies Department, School of Advanced Technologies in Medicine (SATiM), Tehran University of Medical Sciences (TUMS), Tehran, Iran; 7 Department of Biomedical Engineering, University of Connecticut, Storrs, CT, United States of America; University of Maryland at College Park, UNITED STATES

## Abstract

This study aimed to examine the effects of chronic methamphetamine use on the topological organization of whole-brain functional connectivity network (FCN) by reconstruction of neural-activity time series at resting-state. The EEG of 36 individuals with methamphetamine use disorder (IWMUD) and 24 normal controls (NCs) were recorded, pre-processed and source-reconstructed using standardized low-resolution tomography (sLORETA). The brain FCNs of participants were constructed and between-group differences in network topological properties were investigated using graph theoretical analysis. IWMUD showed decreased characteristic path length, increased clustering coefficient and small-world index at delta and gamma frequency bands compared to NCs. Moreover, abnormal changes in inter-regional connectivity and network hubs were observed in all the frequency bands. The results suggest that the IWMUD and NCs have distinct FCNs at all the frequency bands, particularly at the delta and gamma bands, in which deviated small-world brain topology was found in IWMUD.

## 1. Introduction

Methamphetamine (MA) is a highly addictive drug that its consumption is associated with increased feeling of awareness, energy, vigilance, and exhilaration. These psychological effects and relatively easy access have made it a very popular drug among young adults [1]. World Drug Report 2016 reported that there are around 14 to 54 million users of MA worldwide [2]. MA use disorder imposes a large burden on the society; hence, it is important to increase the knowledge about it in the physiological and neurological terms, in order to improve associated diagnosis and treatments.

Functional magnetic resonance imaging (fMRI) and EEG data have been widely used to acquire knowledge about brain disorders such as schizophrenia, Alzheimer’s disease, tinnitus and addiction [3–10]. EEG is portable and less expensive than fMRI. Furthermore, it has a high temporal resolution, which makes it a good instrument to study electrophysiology of the brain in different frequency bands.

So far, few studies have explored the effects of MA use on the brain activity, using resting-state EEG (rEEG) [11]. Newton et al. found increased power at delta and theta oscillatory rhythms in IWMUD compared to the normal NCs [12] and Ahmadlou et al. reported disrupted functional brain organization of IWMUD compared to that of NCs [13].The human brain is a small-world topology which supports both segregation and integration in information processing [14–16]. The network is segregated when containing a large number of connected clusters and integrated when including short path lengths among its units. These characteristics make the network efficient in information transfer with low wiring costs. Previous studies reported that the small-world topology abnormally alters in many disease e.g. major depressive disorder[17], Parkinson's disease [18], Alzheimer’s disease [19], schizophrenia and tinnitus [20, 21], opioids (heroin) and methamphetamine abuse [13, 22], Cirrhosis [23], cognitive disorders related to aging [24, 25].

Ahmadlou et al. have investigated the brain functional organization of IWMUD in early withdrawal stage using functional connectivity network (FCN) and graph theory [13]. They reported increased gamma band small-world index (SWI) in IWMUD using rEEG and constructing the FCN at sensor level.

To date, no study has examined the differences in the brain FCN of IWMUD by rEEG at neuronal source level compared to that of NCs. High-resolution EEG recording combined with source localization methods can provide better spatial resolution for EEG-based connectivity analysis. In the current study, we constructed and compared whole-brain FCN for a group of IWMUD and a group of NCs using rEEG.

## 2. Materials and methods

### 2.1 Participants

Thirty-six IWMUD, with a minimum of 1 and maximum of 6 months of abstinence, were recruited from "Peyrovan Hemmat Harm Reduction Institute" and "Iranian National Center for Addiction Studies (INCAS) Academic Clinic" located in Tehran. 24 age-matched NCs were also recruited in our research. This research is part of a registered brain stimulation trial in Iranian Registry of Clinical Trials (IRCT) in 2018 (IRCT20170808035562N2). The rest EEG were recorded before any intervention. All subjects signed a written informed consent form. Table 1 shows the demographic characteristics and drug use history of the participants.

**Table 1:** Demographic and substance abuse characteristics.

	Descriptive statistics	
	IWMUD	NCs
Gender (male)	36/36	24/24
Age	30.55±6.43	30.75±4.63
Education (years)	14.36±2.79	16.58±2.5
Duration of MA abstinence (months)	1–6	-
Duration of MA use disorder (years)	8.35±4.07	-
Marital status (married)	25/36	7/24
Number of subjects with a history of opium use	20/36	0/24
Number of subjects with a history of alcohol use	23/36	0/24
Number of subjects with a history of heroin use	5/36	0/24
Number of subjects with a history of cigarette smoking	35/36	2/24

### 2.2 EEG data acquisition

We recorded five minutes of rEEG while the participants' eyes were open. We instructed all individuals to pay attention to a black-background screen in front of them during the recording and attempt not to think about anything. All EEG data were recorded using a 62-channel g.tec (http://www.gtec.at/) EEG system (g. HIamp) in National Brain Mapping Laboratory (NBML) (https://nbml.ir/EN). The reference channel was placed on right ear lobe for all individuals. The sampling frequency of 512 Hz was selected for EEG recording. All data were resampled to 200 Hz in preprocessing step to decrease the computational cost.

### 2.3 Data preprocessing

EEG data were preprocessed using EEGLAB [26] and Fieldtrip [27] toolboxes of MATLAB. The datasets were filtered by a 0.1 Hz high-pass filter and a notch filter to remove the voltage drift and 50 Hz power line noise. The data were referenced to common average and artifact rejection was firstly performed by visual inspection. Independent component analysis was employed to remove artifactual components (e.g. eye blinks, eye movements, heartbeat, and muscle artifacts). Then, with a moving window and a peak-to-peak threshold all parts, which exceeded ±100 μv were removed. The preprocessed data, containing the least amount of artifacts, was segmented into 5-second trials (24 trials, totally 120 sec) which were in the range of other resting-state EEG studies [21, 28–30].

### 2.4. Weighted phase lag index (WPLI) description

WPLI is the improved version of PLI connectivity index, proposed by Vinck et al. [27]. It is highly sensitive and powerful to properly detect phase interactions of spatially close signals and has shown robustness to volume conduction that outperforms PLI, coherence, and imaginary coherence (IC) [27, 31, 32]. WPLI estimates the phase leads and lags between two interacted time-series as follows.

$$WPLI_{xy}=\frac{n^{-1}\sum_{t=1}^{n}\left|imag\left(S_{xyt}\right)\right|sgn\left(imag\left(S_{xyt}\right)\right)}{n^{-1}\sum_{t=1}^{n}\left|imag\left(S_{xyt}\right)\right|}$$ (1)

Where S_xyt_ is the cross-spectrum of time-series *x* and *y* at time point *t*, and *sgn* is the sign function. Function imag(.)returns only the imaginary component of the cross-spectrum. WPLI weights the cross-spectrum according to the imaginary component’s magnitude. This allows it to limit the impact of small noise on “true “sign of cross-spectrum around the real axes.

### 2.5 Graph theory analysis

#### 2.5.1. Network construction

After applying Laplacian filter to EEG data to reduce the volume conduction effect and spatially enhance the data quality [33], functional connectivity was computed in EEG-sensor space among pairwise electrodes. The connectivity values were calculated for five EEG frequency bands: delta (1–4 Hz), theta (4–8 Hz), alpha (8–15 Hz), beta (15–30 Hz), and gamma (30–45 Hz) according to previous addiction studies [13, 34]. Accordingly, we obtained a functional network with 61 nodes in five bands (5×61×61 connectivity matrix) for each subject, where the nodes were considered the sensors and the link between them were acquired using the absolute value of the WPLI matrix.

#### 2.5.2 Graph measures

**2.5.2.1 Node strength (NS).** It is sum of the weights of links or edges connected to a node.

$$k_{i}^{w}=\underset{j\in N}{\sum }w_{ij}$$ (2)

Where *N* is the set of all nodes in the network and the links (*i*,*j*) are related by connection weight *w*_*ij*_.

**2.5.2.2 Characteristic path length (CP).** Shortest weighted path length between two nodes *i* and *j* is determined by

$$d_{ij}^{w}=\underset{a_{uv}\in g_{i\overset{w}{↔}j}}{\sum }f\left(w_{uv}\right)$$ (3)

where *f* is a map (e.g. an inverse) from weight to length and $g_{i\overset{w}{↔}j}$ indicates the shortest weighted path between nodes *i* and *j*. The averaged shortest path length between all the node pairs in a network is known as the characteristic path length [35]:

$$CP^{w}=\frac{1}{n}\underset{i\in N}{\sum }\frac{\sum_{j\in N,j\neq i}d_{ij}^{w}}{n-1}$$ (4)

where *n* shows the number of nodes.

**2.5.2.3 Clustering coefficient (CC).** The number of weighted triangles around a node *i* is defined as a basis for measuring segregation:

$$t_{i}^{w}=\frac{1}{2}\underset{j,h\in N}{\sum }{\left(w_{ij}w_{ih}w_{jh}\right)}^{\frac{1}{3}}$$ (5)

Clustering coefficient reflects the degree that the connected nodes in a graph tend to form clusters and can illustrate the degree of local connectivity in the network [35, 36]. The clustering coefficient of the network is described by:

$$CC^{w}=\frac{1}{n}\sum_{i\in N}cc_{i}^{w}=\frac{1}{n}\sum_{i\in N}\frac{2t_{i}^{w}}{k_{i}\left(k_{i}-1\right)}$$ (6)

Characteristic path length measures the integration of the network, while the clustering coefficient is a measure for the network functional segregation.

Next to C and L, by following previous studies [13, 37], the small-world index (*SWI* = *CC*^*w*^/*CP*^*w*^) was obtained for each individual. The larger the SWI value is, the more small-world the network is. The small world organizations have simultaneously notably segregated and integrated topologies [14, 15].

#### 2.5.3 Hub identification

Hubs refer to highly linked nodes in the network. Following the method used in previous studies [38] we used the node strength, betweenness centrality [14] and eigenvector centrality [14] to identify hubs using BCT toolbox [14]. Nodes in each of the mentioned measures that exceed one standard deviation from the mean value of the measure were considered as hubs. Once the hubs were identified using the different techniques, those hubs that were commonly obtained by the different techniques were compared between MA abusers and controls.

### 2.6. Source reconstruction

Standardized low-resolution brain electromagnetic tomography (sLORETA) [39] was used to estimate the intracerebral electrical sources, using FieldTrip. sLORETA computes neuronal activity in current density (A/m^2^) without assuming a predefined number of active sources. We first acquired a lead field (forward model) by creating a FEM volume conduction model of the head. To do this, we used 61 electrodes, a grid with 3 mm^3^ resolution, and voxels of the anatomical MRI (colin27 brain), segmented (i.e. separated) into the five different tissue types: scalp, skull, CSF (Cerebro-Spinal Fluid), gray and white matter. We used sLORETA to reconstruct neuronal activities in source points in the gray matter (cortical regions).

To parcellate the brain into 90 (45 in each hemisphere) regions of interest (ROIs), automated anatomical labeling (AAL) atlas were applied [40]. Table 2 lists the name of the ROIs and their corresponding abbreviations. The single nearest voxel to central voxel has been considered as the great representation of each ROI by following previous studies [41, 42]. Accordingly, we obtained a functional network with 90 nodes in the five frequency bands (5×90×90 connectivity matrix) for each subject, where the nodes are equivalent to the central points of ROIs of AAL template and the link between them are the absolute value of the WPLI matrix. MNI coordinates of the selected central voxels are brought in S1 Table.

**Table 2:** The names and the corresponding abbreviations of the ROIs specified in the AAL brain template (45 regions for each hemisphere) described by Tzourio-Mazoyer et al.[40].

Regions	Abb.	Regions	Abb.
Precentral gyrus	PreCG	Lingual gyrus	LING
Superior frontal gyrus (dorsal)	SFGdor	Superior Occipital gyrus	SOG
Orbitofrontal cortex (superior)	ORBsup	Middle occipital gyrus	MOG
Middle frontal gyrus	MFG	Inferior occipital gyrus	IOG
Orbitofrontal cortex (middle)	ORBmid	Fusiform gyrus	FFG
Inferior frontal gyrus (opercular)	IFGoperc	Postcentral gyrus	PoCG
Inferior frontal gyrus (triangular)	IFGtriang	Superior parietal gyrus	SPG
Orbitofrontal cortex (inferior)	ORBinf	Inferior parietal lobule	IPL
Rolandic operculum	ROL	Supramarginal gyrus	SMG
Supplementary motor area	SMA	Angular gyrus	ANG
Olfactory	OLF	Precuneus	PCUN
Superior frontal gyrus (medial)	SFGmed	Paracentral lobule	PCL
Orbitofrontal cortex (medial)	ORBmed	Caudate	CAU
Rectus gyrus	REC	Putamen	PUT
Insula	INS	Pallidum	PAL
Anterior cingulate gyrus	ACG	Thalamus	THA
Middle cingulate gyrus	DCG	Heschl gyrus	HES
Posterior cingulate gyrus	PCG	Superior temporal gyrus	STG
Hippocampus	HIP	Temporal pole (superior)	TPOsup
Parahippocampal gyrus	PHG	Middle temporal gyrus	MTG
Amygdala	AMYG	Temporal pole (middle)	TPOmid
Calcarine cortex	CAL	Inferior temporal gyrus	ITG
Cuneus	CUN		

## 3.Statistical analysis

We used the most relevant study carried out by Ahmadlou et al. to obtain the effect size for most important variable [13]. As in that study, SWI in the gamma frequency band, which is a ratio of CC and CP, has been suggested as a potential bio-marker for IWMD who are in early stage of MA withdrawal (<1 month), we considered it as the most important variable, primary endpoint, to assess it for IWMD who are in middle stage of MA abstinence (>1 month).

We obtained an effect size of 0.96 and yielded a size of 18 in each group with type I error 5% (α = 0.05) and study power 80% (β = 0.2) using two-tailed test and G*Power software.

A multivariate analysis of variance (MANOVA) model was used to investigate statistical differences between topological metrics of brain FCN in IWMD and NCs. The model assumptions were checked to be held: multivariate normality by Shapiro-Wilk test; homogeneity of covariance matrices by Box’s M test; homogeneity of variance by Levene’s test; further, the absence of multicollinearity was checked by variance inflation factor. The effects of potential baseline confounding variables, including age and total-score of depression, anxiety and stress (DASS-21 scale) were controlled in the model. There was no any missing variable in the twenty topological characteristics but there were a few ones for the DASS-21 scale that have been replaced by mean imputation.

Among the twenty variables, just six variables met the assumptions. Hence, the MANOVA in sensor and source spaces comprised six dependent variables (DVs) and one independent variable with two levels (IWMD vs. NCs). For the remaining variables, non-parametric test (Mann–Whitney) was used. An *α* level of less than 0.05 was considered significant. The analysis was carried out using “SPSS 22”.

To control the type I error in multiple comparisons of connectivity differences in each frequency band, Benjamini–Hochberg procedure was carried out with false discovery rate 0.05 (q-value<0.05). The analysis was performed using MATLAB software.

## 4. Results

### 4.1 Sensor space results

MANOVA showed that there was no statistically significant difference in DVs based on group levels (IWMD and NCs), F (1, 58) = 0.83, p = 0.54; Wilk's Λ = 0.9. Adjusting for potential confounders including DASS and age had no significant effect on this association.

Mann–Whitney test revealed that NS (U = 224,p = 0.002) and SWI(U = 225, p = 0.002) of IWMD in the delta frequency band is statistically significantly higher than those of NCs. Further, CP of IWMD in delta frequency band are statistically significantly lesser than those of NCs, (U = 246,p = 0.007), (Table 3).

**Table 3:** The statistical differences of topology metrics between IWMD and NCs (Mann–Whitney *U* / p-value) or (F/p-value) in sensore space.

	delta	Theta	alpha	beta	gamma
NS	**224/ 0.002**	345/ 0.24	419/0.9	331/0.17	331/0.17
CC	323/0.13	338/0.2	0.56/0.4^a^	0.002/0.9^a^	0.9/0.7^a^
CP	**246/0.007**	1.9/0.1^a^	0.003/0.9^a^	322/0.13	1.6/0.2^a^
SWI	**225/0.002**	325/0.14	362/0.37	392/0.6	354/0.3

The ‘^a^’ indicates parametric test. Significant differences (p<0.05) are bold.

**Fig 1:**
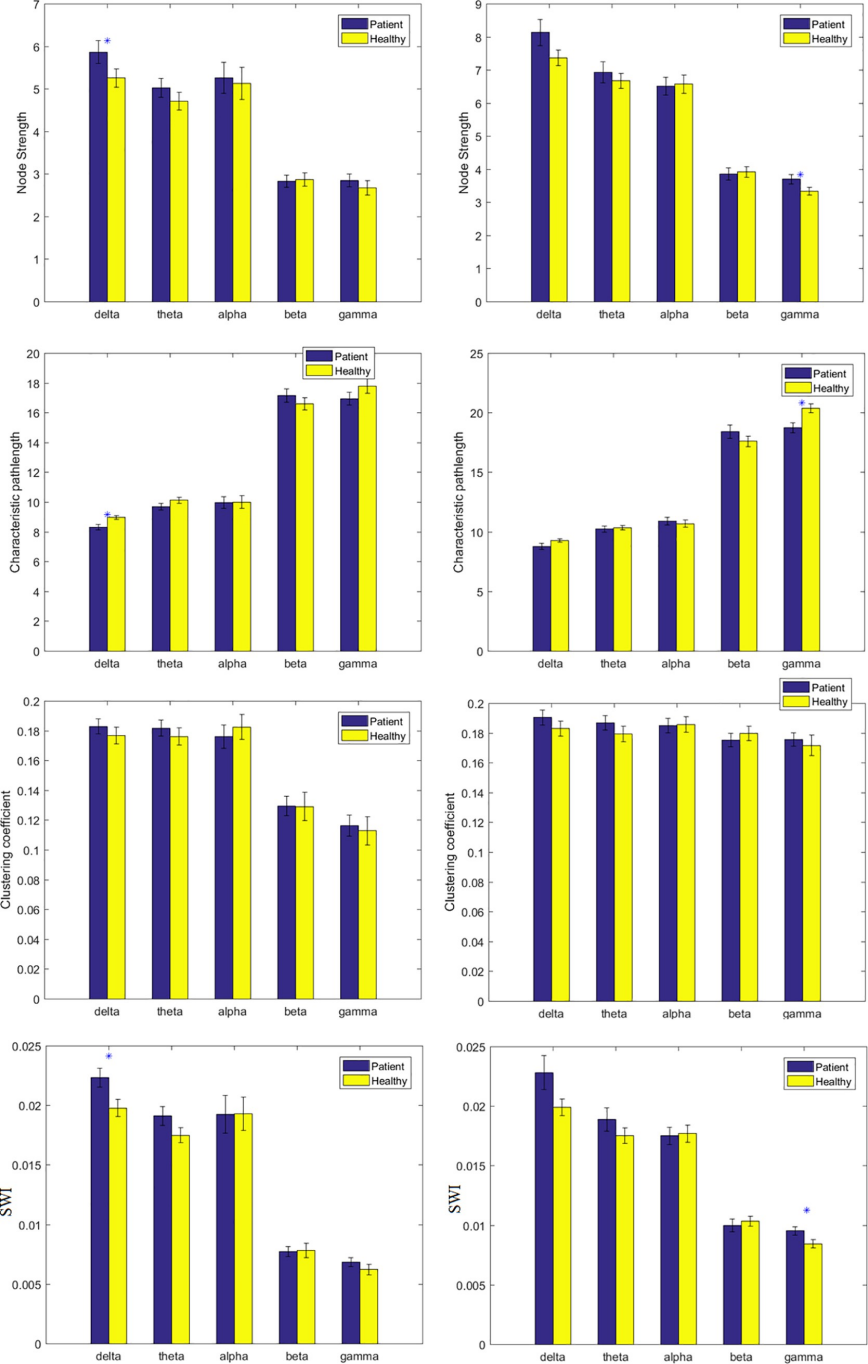
Left column: Computed topological metrics (NS, CC, CP, and SWI) in the sensor space. Right column: computed topological metrics in the source space. If the p-value is less than 0.05 it is flagged with one star (*). Each bar represents mean values ±SE.

The mean and SD of the topological metrics at all the frequency bands are brought in Table 4 and shown in Fig 1. The values of topological metrics in sensor space along with related statistical log file are brought in S2 Table and S1 File.

**Table 4:** Mean and standard deviation of the brain topology metrics in five frequency bands for IWMUD and NCs acquired in sensor space.

Frequency Band	NS	CC	CP	SWI
	Mean(SD) Patient/Control	Mean(SD) Patient/Control	Mean(SD) Patient/Control	Mean(SD) Patient/Control
**Delta**	5.8663 (1.6133) / 5.2585 (1.0389)	0.18299 (0.030608) / 0.17685 (0.026965)	8.34(1.06)/ 8.9861 (0.64487)	0.2235(0.00357)/ 0.019796 (0.0034958)
**Theta**	5.0234 (1.3399) / 4.7144 (1.0519)	0.18181 (0.032516) / 0.1763 (0.028919)	9.73 (1.29) / 10.1423 (1.0025)	0.01913(0.003938)/ 0.017494 (0.0034958)
**Alpha**	5.2648 (2.1662) / 5.1363 (1.8444)	0.17625 (0.047435) / 0.18263 (0.040771)	9.97 (2.29) / 10.0138 (2.1707)	0.01926(0.008890)/ 0.019305 (0.0034958)
**Beta**	2.8326 (0.84394) /2.8701 (0.75815)	0.12963 (0.038471) / 0.12923 (0.046343)	17.16 (2.73) / 16.6225 (2.0136)	0.0077(0.00240)/ 0.0078475 (0.0034958)
**Gamma**	2.8529 (0.88257) /2.6812 (0.83643)	0.11636 (0.04189) / 0.11292 (0.046492)	16.95(2.62)/ 17.8008 (2.3357)	0.0068(0.00194)/ 0.00624 (0.0034958)

### 4.2 Source space results

The MANOVA showed that there was no statistically significant difference in DVs based on group levels (IWMD and NCs), F (1, 58) = 1.13, p = 0.35; Wilk's Λ = 0.88. Adjusting for potential confounders including DASS and age had no significant effect on this association.

Mann–Whitney test revealed that NS (U = 254,p = 0.007) and SWI (U = 284, p = 0.02) of IWMD in the gamma frequency band are statistically significantly higher than those of NCs (Table 5).

**Table 5:** The differences of all topology metrics between IWMD and NCs (Mann–Whitney *U* / p-value) or (F/p-value) in source space.

	delta	theta	alpha	beta	gamma
NS	338/0.15	412/0.7	0.032/0.8^a^	322/0.09	**254/0.007**
CC	365/0.3	1.8/0.178^a^	0.024/0.8^a^	0.96/0.3^a^	416/0.8
CP	2.3/0.13^a^	422/0.8	0.2/0.6^a^	323/0.1	**269/0.01**
SWI	325/0.1	371/0.3	393/0.5	331/0.1	**284/0.02**

The ‘^a^’ indicates parametric test. Significant differences (p<0.05) are bold.

The mean and SD of the topological measurements at all the frequency bands are brought in Table 6 and shown in Fig 1. The values of topological metrics in source space along with related statistical log files are brought in S3 Table and S2 File.

**Table 6:** Mean and standard deviation of the brain topology metrics in five frequency bands for IWMUD and NCs acquired in source space.

Frequency Band	NS	CC	CP	SWI
	Mean(SD) Patient/Control	Mean(SD) Patient/Control	Mean(SD) Patient/Control	Mean(SD) Patient/Control
**delta**	8.1383 (2.3436) / 7.3786 (1.1594)	0.19055 (0.030569) / 0.18306 (0.024361)	8.7826 (1.45) / 9.2687 (0.72281)	0.0228 (0.008) / 0.019924 (0.0033749)
**theta**	6.9361 (1.9247) / 6.6823 (1.107)	0.1868 (0.029246) / 0.17952 (0.025357)	10.24 (1.53) / 10.3356 (0.90367)	0.0189 (0.0055) / 0.01754 (0.0033749)
**alpha**	6.5183 (1.5906) / 6.5775 (1.3634)	0.18508 (0.028833) / 0.18584 (0.026051)	10.91 (1.93) / 10.6937 (1.4524)	0.0175 (0.0039) / 0.017707 (0.0033749)
**beta**	3.86 (1.1416) / 3.9243 (0.78829)	0.17541 (0.026241) / 0.17984 (0.024427)	18.43 (3.43) / 17.6271 (2.17)	0.0099 (0.0029) / 0.010369 (0.0033749)
**gamma**	3.7041 (0.83654) / 3.338 (0.57717)	0.17577 (0.027282) / 0.17171 (0.033844)	18.76 (2.50) / 20.402 (1.8259)	0.0095 (0.001728826) / 0.0084513 (0.0033749)

**Fig 2:**
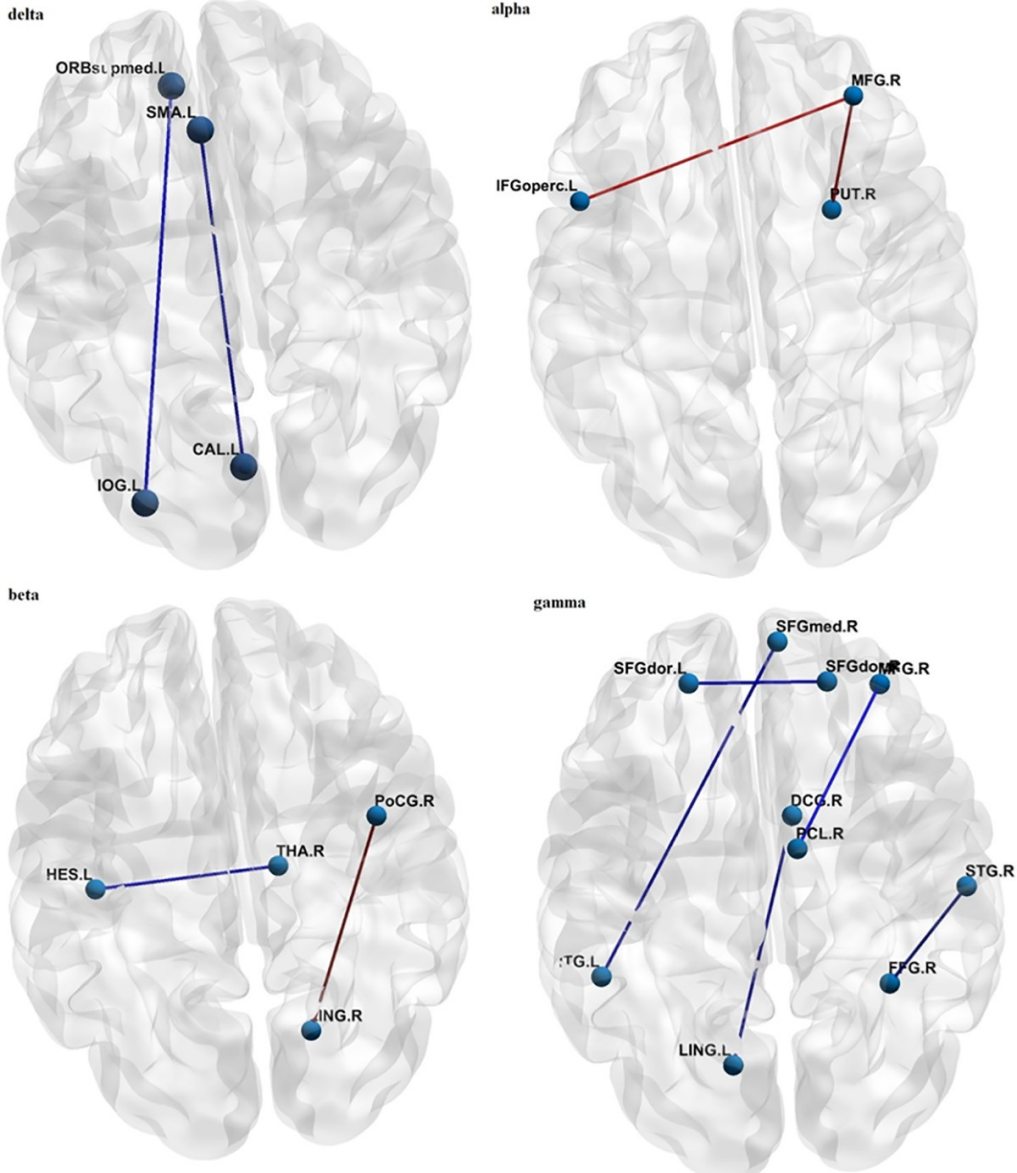
Functional connectivity differences after FDR correction in the frequency bands. The red/blue means attenuated/enhanced inter-regional connectivity in IWMUD compared to NCs.

The functional connectivity values were significantly different between two groups for some pairs of AAL regions at all the frequency bands except at the theta band. In this regard, the significance level was α = 0.05 using false discovery rate (FDR) q<0.05 to correct for multiple comparisons. The gamma oscillatory rhythm revealed more connectivity differences compared to the other rhythms. At the gamma band range, the functional connectivity of IWMUD was greater than those of NCs, in seven AAL pairs, while for the delta band only two pairs have shown enhanced coupling values in IWMUD compared to those of NCs. At the alpha frequency band, IWMUD showed just attenuated functional coupling in two AAL pairs compared two NCs, while at the beta oscillatory rhythm both enhanced and attenuated functional couplings were revealed. There were no significant differences between two groups in the theta band. These coupling differences are mapped on the brain image in Fig 2 using BrainNet Viewer [43].

**Fig 3:**
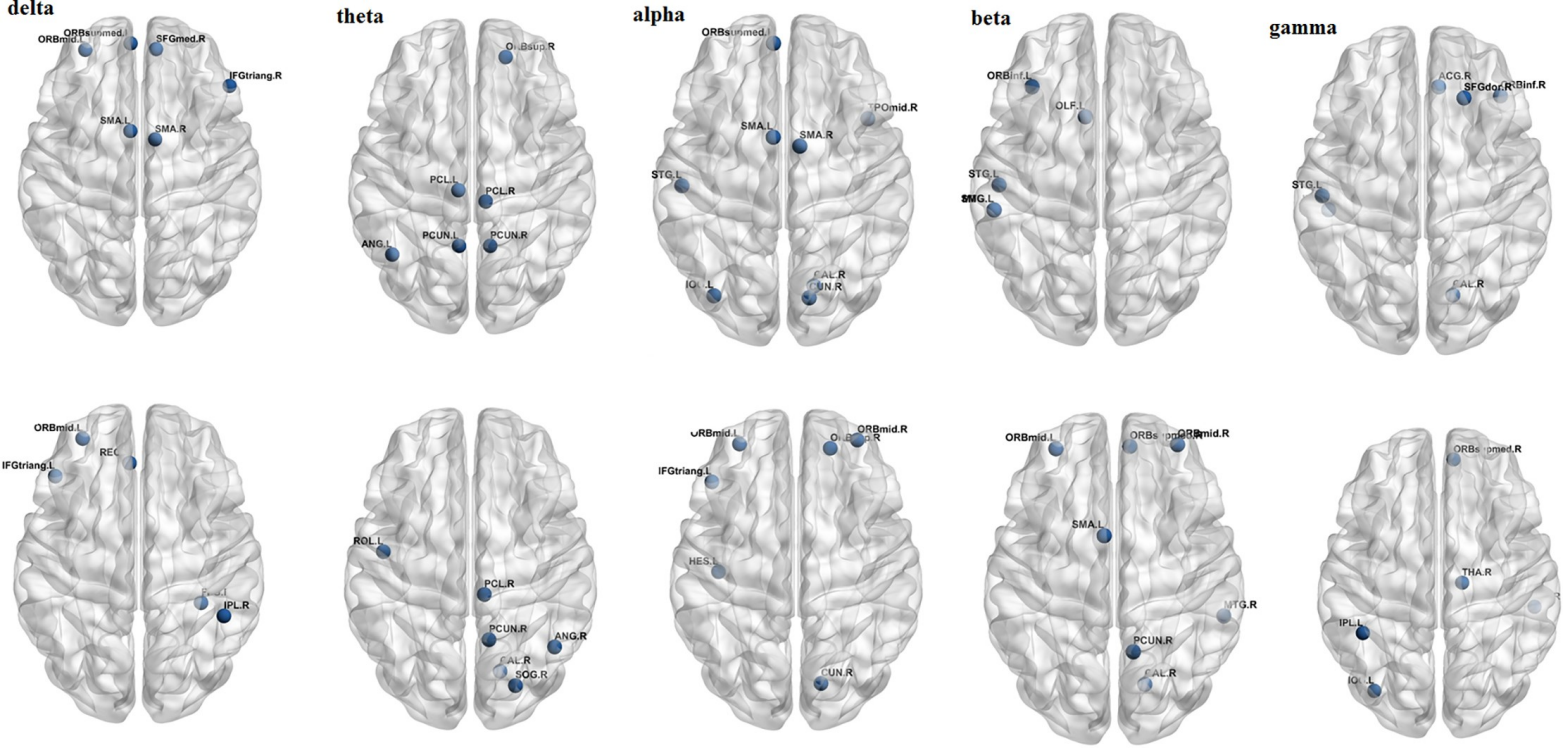
Hubs of IWMUD (top row) and NCs (bottom row) in the five frequency bands. The figures are ploted using brain net.

Common hubs calculated from the FCN of the two groups using the centrality measures were different in the five frequency bands. These hubs are listed in Table 7 and mapped on the brain in Fig 3.

**Table 7:** The name of specific hubs of patient and control and common hubs between the two groups.

**IWMD**	**delta**	Frontal_Mid_Orb_L	Frontal_Inf_Tri_R	Supp_Motor_Area_L	Supp_Motor_Area_R	Frontal_Sup_Medial_R	Frontal_Med_Orb_L		
	**theta**	Frontal_Sup_Orb_R	Angular_L	Precuneus_L	Precuneus_R	Paracentral_Lobule_L	Paracentral_Lobule_R		
	**alpha**	Supp_Motor_Area_L	Supp_Motor_Area_R	Frontal_Med_Orb_L	Calcarine_R	Cuneus_R	Occipital_Inf_L	Temporal_Sup_L	Temporal_Pole_Mid_R
	**beta**	Frontal_Inf_Orb_L	Olfactory_L	SupraMarginal_L	Temporal_Sup_L	Temporal_Mid_L			
	**gamma**	Frontal_Sup_R	Frontal_Inf_Orb_R	Cingulum_Ant_R	Calcarine_R	Temporal_Sup_L	Temporal_Inf_L		
**NCS**	**delta**	Frontal_Mid_Orb_L	Frontal_Inf_Tri_L	Rectus_L	Fusiform_R	Parietal_Inf_R			
	**theta**	Rolandic_Oper_L	Calcarine_R	Occipital_Sup_R	Angular_R	Precuneus_R	Paracentral_Lobule_R		
	**alpha**	Frontal_Sup_Orb_R	Frontal_Mid_Orb_L	Frontal_Mid_Orb_R	Frontal_Inf_Tri_L	Cuneus_R	Heschl_L		
	**beta**	Frontal_Mid_Orb_L	Frontal_Mid_Orb_R	Supp_Motor_Area_L	Frontal_Med_Orb_R	Calcarine_R	Precuneus_R	Temporal_Mid_R	
	**gamma**	Frontal_Med_Orb_R	Occipital_Inf_L	Parietal_Inf_L	Thalamus_R	Temporal_Inf_R			
**Common Hubs**	**delta**	Frontal_Mid_Orb_L							
	**theta**	Precuneus_R	Paracentral_Lobule_R						
	**alpha**	Cuneus_R							

### 4.3 Results of self-reported measurements

We used Barratt Impulsiveness Scale-11 (BIS-11) and Depression Anxiety Stress Scale-21 (DASS-21) to measure self-reported impulsivity, depression, anxiety and stress. The anxiety and stress values were significantly different between the two groups (P<0.001), but the key variable, SWI in the gamma frequency band, was not significantly correlated with these self-reported scales (Table 8).

**Table 8:** Results of correlations between anxiety, depression, stress and impulsivity and the SWI values in the gamma band for the patient and control groups.

		Mean (SD)		Z	P-value	Corr. Coef. / P-Value	
Characteristic	Subjects (IWMD/NCS)	IWMD	NCs			IWMD	NCs
**Stress**	30/21	19.66(10.62)	6.10(2.7)	-4.7	<0.001	-.16/ 0.37	-0.07/0.75
**Anxiety**	31/23	12.90 (8.66)	5.5(5.6)	-3.4	.001	0.02/ 0.92	-0.06/0.78
**Depression**	29/23	18.34(10.14)	14.2(6.4)	-1.4	0.15	-0.008/ 0.96	-0.12/0.58
**Total DASS**	36/24	46(21)	26(12)	-4.2	<0.001	-0.17/0.47	-0.09/0.65
**Attention impulsivity**	36/-	12.11(4.81)				0.15 / 0.35	
**Motor impulsivity**	33/-	15.3(6.17)				0.38/ 0.03	
**Nonplanning impulsivity**	35/-	17.62 (4.09)				-0.08 / 0.6	

The missing values of Total DASS were replaced by mean series. Spearman correlation was used.

### 4.4 Power results

**Fig 4:**
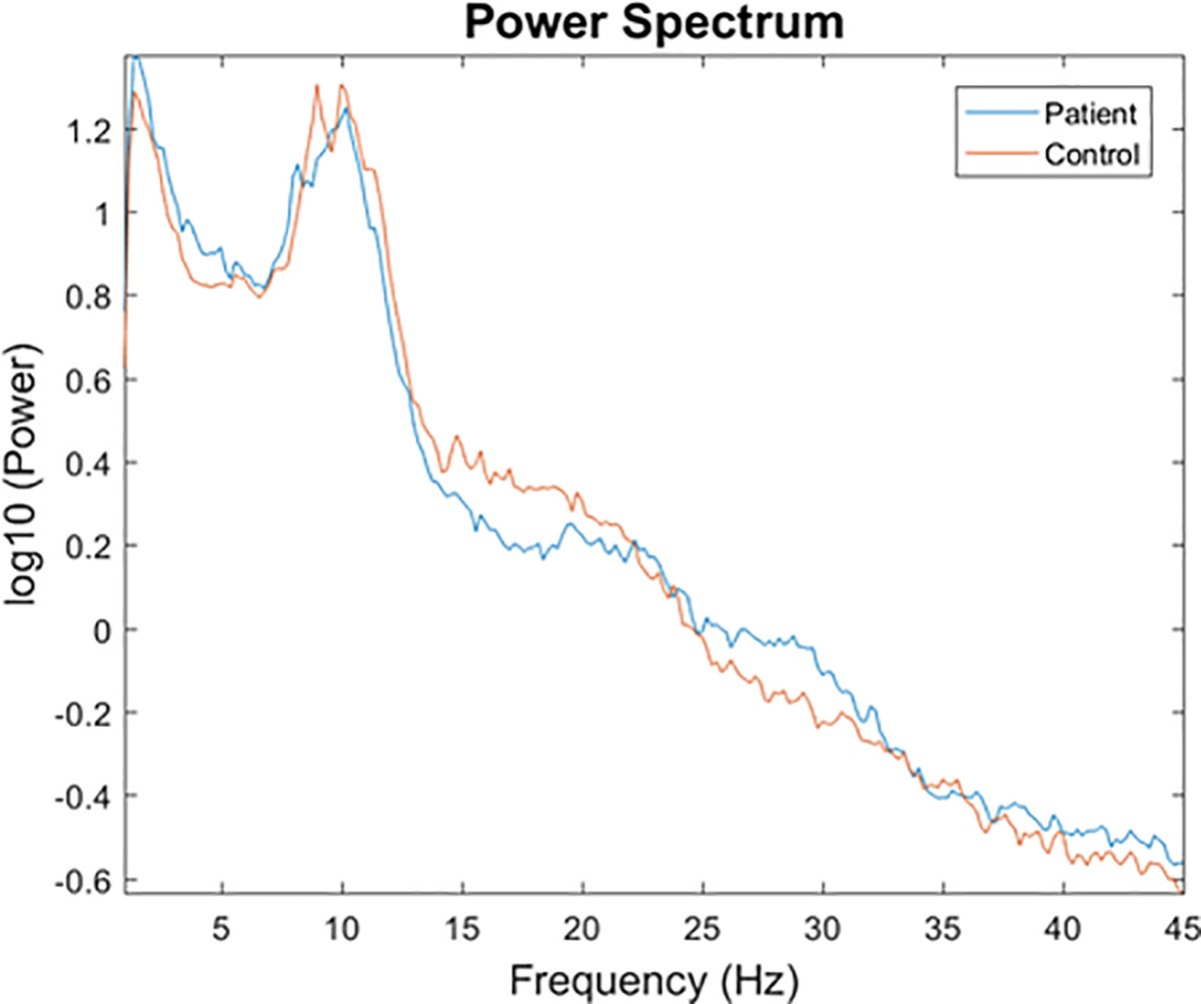
The power spectrum of IWMUD and NCs computed in neural source space.

Power analysis revealed no significant differences between IWMUD and NCs at all the frequency bands. Fig 4 shows the power spectrum of the two groups.

## 5. Discussion

In this study, we compared brain FCN of IWMUD with that of NCs using graph features. To our knowledge, this is the first study that assessed FCN of IWMUD at neuronal source level using rEEG. Table 9 summarizes the main findings of the current study along with previous related studies.

**Table 9:** Meta data of previous studies who recruited MA abusers with corresponding data of the current study.

Study	Subjects (number of males)	Age (year) mean (SD)	Use Duration (year)	Abstinence Duration (days)	Analysis space	Main Findings
	IWMD/NCs	IWMD/NCs	Mean (SD)	Mean (SD)	EEG sensors	
Newton et al. [12]	11/11 (8/8)	32.7 (7.5)/36.5 (7.3)	11.0 (3.5)	4 (0)	EEG sensors	Enhanced delta and theta power
Yun et al. [44]	48/20 all males	37.0 (5.8) / 34.5 (7.7)	11.8 (6.5)	30.5 (27.2)	EEG sensors	Decreased cortical complexity
Khajehpour et al. [45]	36/24 all males	30.55 (6.43)/ 30.75(4.63)	8.35(4.07)	Range 30 to 180	EEG sensors	Automatic discrimination of IWMD from NCS (F-score = 0.94)
Ahmadlou et al.[13]	36/36 all males	31.7 (8.8)/32.7 (6.8)	6.42 (3.13)	Range 7 to 21	EEG sensors	Disrupted functional brain topology at gamma band
The Current study	36/24 all males	30.55(6.43)/ 30.75(4.63)	8.35(4.07)	Range 30 to 180	Neural sources	Disrupted functional brain topology in delta and especially in gamma bands. Altered inter-regional connectivity in delta, alpha, beta and gamma bands. Altered hub pattern in all the frequency bands.

### 5.1 Functional connectivity alterations

EEG oscillatory rhythms have been related to specific functions exclusively or in a combination. In this context, studies show that gamma band frequencies are associated with perception, attention, stimulus selection, memory process and conscious awareness [46–52]. The theta rhythms have been related to attention, working memory, and emotional arousal [53]. The delta frequencies have been linked to learning motivation, memory and reward processing, while alpha-band oscillations that are the dominant oscillations in the human brain, has been linked to working memory functions [52, 54, 55]. Beta-band activity is related to cognition [56, 57]. A recent study reported that multiple oscillatory rhythms determine the temporal organization of perception [56]. Hence, abnormal oscillations have been related to brain specific dysfunction. For example, abnormal gamma oscillations, have been related to dysregulation of the dopaminergic system in the diseases of central nervous system, disinhibition in GABAergic system, excitatory activation of the brain, and drug-seeking behaviors in addiction [58–60]. Task-based fMRI studies showed that there are six networks abnormally changed in addiction [7, 61]: default mode network (DMN), salience network, habit network, executive control network and memory network. These networks revealed hyper-activation during drug cue exposure compared to neutral stimuli. Zilverstand et al. [61], in accordance with impaired response inhibition and salience attribution (iRISA) model [62], proposed increased engagement of these networks to cognitive drug cue processing in addiction. The Resting-state fMRI (rfMRI) studies reported that in chronic stimulant users, the reward, salience, habit, and memory networks demonstrated enhanced coupling with each other, as well as with the executive network, whereas a decreased coupling was observed within the executive control network [61].

In the current study, neural source level connectivity revealed enhanced intra-connectivity at the gamma band within DMN network (Frontal-superior-R/L, frontal superior medial_R, Temporal_inf_L), improved inter-connectivity between DMN and visual network (cingulum-mid-right and ligual_L), also between executive control and sensorimotor networks (frontal-mid right, Paracentral-lobule_R). However previous resting-state studies did not find increased intra-connectivity within DMN, related results of this study are in line with those of fMRI studies with drug cue presentation [61]. The enhanced coupling between the executive network and sensorimotor is consistent with previous studies [61]. Taken together, the abnormally increased coupling at the gamma band may imply disrupted cognitive control regarding attention and self-monitoring that may be interpreted in the perspective of altered attention toward drug related stimuli.

Furthermore, the correlation between Putamen, which is an impaired region of habit network in addicts [61], and impulsivity in IWMUD at the gamma band may be another evidence for the addictive behavior.

Evidence reveals that the left inferior temporal gyrus may be involved in retrieval load and visual perception [63–65]. Task-based and rfMRI studies have reported abnormally increased activity of left inferior temporal gyrus. High-risk college students showed hyper-activation of this gyrus during exposure to poly-drug cues compared to neutral cues [66]. Cocaine-dependent individuals encountered with increased activity of their left inferior temporal gyrus in the reward in comparison to no-reward condition [67]. Amphetamine-type Stimulant abusers showed increased degree centrality in the left inferior temporal gyrus in a rfMRI study [68]. In the current study, left inferior temporal gyrus showed increased node strength at the gamma band in IWMUD compared to NCs. Hence, we may be able to conclude that the IWMUD may allocate more attention resources to think about obtaining and using methamphetamine, reflected in increased node strength of the left inferior temporal gyrus at the gamma oscillatory band.

Comprehensive systematic reviews on resting-state brain patterns proposed that salience and executive networks are tightly connected during acute drug use, while they become less connected during abstinence. They further suggested that disengagement of the two networks during abstinence impairs non-drug-related processing [7]. We found consistent results with this disengagement at the alpha frequency band. In this regard, our results revealed decreased intra-coupling in executive network (Frontal Mid-R and Frontal-Inf-Oper-L), which is consistent with rfMRI findings [61], and inter-coupling between executive and salience networks (Putamen-R and Frontal-Mid-R) that by following the reasoning of [7, 61] could lead to the disengagement of salience and executive networks during abstinent and impairs non-drug-related processing in IWMUD. Although, at the gamma band the MA’s brain undergoes no decreased coupling, and this contrary-frequency alteration result requires further research using EEG to speculate why it is occurring.

The occipital lobe is the visual processing center of the brain and Medial-OFC is related to the reward system that has shown abnormally increased connectivity during drug cue exposure in substance abusers[61]. Hence, although resting-state were investigated, the findings of abnormally increased connectivity of medial-OFC and occipital lobe at the delta band may be related to deficits in behavioral inhibition in IWMUD. Nevertheless, due to EEG high-pass filtering influence on the connectivity values at this oscillatory band, we should interpret the delta-related results cautiously.

### 5.2 Topological characteristic alterations

The present study examined the topological organization of the functional brain networks in IWMUD compared to NCs. The human brain is a complex system, with important topological attributes such as small-world property, high clustering coefficient, and small characteristic path length. These traits yield a highly integrated and segregated networks which are efficient for information transfer [36, 69].

The source level results revealed that IWMD had significantly increased SWI and S with decreased L at the gamma band compared to NCs. These results are in line with those of Ahmadlou et al. [13]. This implies enhanced integration and segregation of FCN among IWMUD at the expense of increased node strength at the gamma frequencies.

The majority of connectivity changes at the gamma band are attributed to DMN network. Hence, one may speculate that the gamma-dependent small-worldness may reflect an alteration in the brain functional topology of IWMUD to be more efficient in an inter-modal dysfunctional network associated with rumination or other maladaptive self-referential propensities.

Similar differences were also revealed in the delta band: significantly increased C, increased L and SWI. Ahmadlou et al. also found a small difference in C at this frequency band. Therefore, it could be concluded that brain dysfunction at the delta and gamma frequency band in IWMUD is expected.

Balcony et al. suggested that the delta frequencies may be responsive-relevant rewarding cues and their modulation may be related to a reward bias [70]. A meta-analysis on fMRI studies [71] suggested that the visual cortex consistently discriminates drug cues from neutral cues in substance-dependent populations. The result of delta band revealed increased connectivity between visual cortex (Occipital_Inf_L) and executive network (Frontal_Med_Orb_L). Hence, it may be related to the attribution of incentive salience to drugs and drug-associated cues in IWMUD.

Despite the differences in results at sensor and source level, however, their trend is the same and it could be attributed to the fact that each EEG electrode collects the sum of the electrical activity from different sources. Also, the source-localization is a statistical estimation of the sources and, therefore, is subject to technical limitations for accurate estimation. Filtering may cause some other aberrant differences between connectivity values of the two groups [72]. To decrease the notch filtering influence at the gamma band, we examined two frequency ranges, 30-45Hz by following previous studies [34, 73] and 30-60Hz similar to the work presented at [13]. The first range that is preserved from notch filtering effect yielded no significant differences by the sensor space analysis, while the second range (30–60 Hz) led to significant results at the gamma band that may be due to notch filtering influence. Hence, here, the range of 30–45 Hz was selected to attenuate the likely disruptive effect of notch filtering on connectivity.

Brain disorders are related to altered brain coupling that reflects either as a complete variation in the network topology by the replacement of hubs or by alteration of their inter connectivity [74]. According to the hub-associated findings, it appears that the brain FCN of IWMUD and NCs could be considerably distinct, not only at the delta and gamma band but also at the other oscillatory bands because of having few common hubs in all the oscillatory bands between IWMUD and NCs.

Gamma-related hubs of IWMUD are mostly attributed to reward (Frontal_Inf_Orb_R), salience (Cingulum_Ant_R), DMN (Frontal_Sup_R, Temporal_Inf_L), visual and auditory (Calcarine_R and Temporal_Sup_L), and the delta-associated hubs are mostly located in reward (Frontal_Mid_Orb_L, Frontal_Med_Orb_L), DMN (Frontal_Sup_Medial_R) and sensorimotor network.

Activations of sensorimotor areas in response to drug stimuli are correlated to craving, the severity of dependence and automatized behavioral reactions towards drug-related stimuli [75]. Salience and reward networks are also important in this regard. In sum, it may be speculated that hub replacement and connectivity alterations in the gamma and delta frequency bands are associated with regions that have an important role in disability of substance-dependent individuals to control their addiction-related behaviors.

The delta and gamma related topology and connectivity changes could facilitate the development of new treatment strategies and serves as a predictive biomarker of disease severity and treatment outcome for IWMUD.

The recruited IWMUD of the current study were in their middle stages of abstinence (1–6 months) and they did not experience considerable withdrawal effects. Therefore compared to participants of Ahmadlou’s study [13] who were in early withdrawal stage (< 3 weeks), probability of this hypothesis is reinforced that the dysfunctional organization of the gamma frequency band indexed by abnormal small-world properties of the brain FCN may be associated with chronic exposure to MA and not to the abstinence.

### 5.3. EEG activity and impulsiveness, depression, anxiety and stress scales

According to a study among individuals with gambling disorder [76], high impulsivity (25th percentile of BIS-11 scale) has the potential to affect EEG power spectrum in different frequency bands except for the gamma band. Stress may alter EEG waves so that the baseline rhythms in individuals with mild and moderate stress is alpha wave and in those with high stress is beta wave [77]. Our recruited subjects were mostly in normal, mild or moderate levels of impulsivity, stress, anxiety, and depression (Table 10). The correlation analysis revealed no significant correlation between the gamma SWI and impulsivity or DASS scales; further, the DASS score showed no significant effect in the MANOVA model. Moreover, previous related study also reported abnormal alteration of brain topology metrics in this frequency band [13]. Accordingly, it may be concluded that the findings in the gamma frequency band are probably due to methamphetamine dependence and its effect on brain resting-state networks, not withdrawal effects. Nevertheless, the findings of other frequency bands may be affected by the impulsivity and stress levels and also the power of brain activity is not directly associated with its functional connectivity. We could not asses these undesired effects because the sample size was low and just a few variables had the eligibility to be included in the MANOVA. Conducting a related research with large sample size is therefore suggested to confirm the functional alterations found in the alpha and theta frequency bands.

**Table 10:** Number of subjects in different stress, anxiety, and depression levels.

		Normal	Mild	Moderate	High	Sever
IWMD	Stress	12	2	8	3	5
	Anxiety	9	3	8	4	7
	Depression	5	6	7	4	7
NCs	Stress	21	0	-	-	-
	Anxiety	16	4	1	1	1
	Depression	3	8	10	0	2

### 5.4 Limitations

The present study had several limitations. First, as a human study, there were some intrinsic limitations from matching the behavioral and demographic characteristics of IWMUD and NCs because of inaccessibility of backgrounds of the participants. Therefore, more accurate research using genetically modified animals with better control matching are required to support the obtained results of this study. Second, as some factors could probably vary the developmental period of vulnerability to methamphetamine toxicity in different genders, we recruited only male participants to remove the confounding factor of gender variations [78, 79]. Research including both genders is needed. Third, we managed to scan the IWMUD during substance withdrawal, while the NCs were not, so we were not able to absolutely exclude the influence of smoking, drinking or caffeine. Forth, the numbers of subjects were moderate because of practical difficulties in recruitment of IWMUD. Fifth, subcortical network (Putamen, palladium, thalamus, caudate) cannot be properly reconstructed by EEG.

## 6. Conclusion

Inter-regional functional connectivity and topological characteristics of brain FCN in IWMUD are abnormally changed in the delta and gamma oscillatory bands.

Nevertheless, it seems that brain dysfunction of IWMUD is not limited to these frequency bands, as altered hub patterns are extended to all the oscillatory rhythms. These findings may help provide predictive biomarkers of disease severity and treatment outcome for IWMUD.

## Supporting information

S1 Table MNI coordinates of the central voxel of the ninety AAL regions. (XLSX)

S2 Table Value of the BCN topology metrics for all subjects computed in sensor space. (XLSX)

S3 Table Value of the BCN topology metrics for all subjects computed in source space. (XLSX)

S1 File SPSS file (.spv format) of statistical analysis in sensor space. (RAR)

S2 File SPSS file (.spv format) of statistical analysis in source space. (RAR)

## References

[1] Noroozi A, Malekinejad M, Rahimi-Movaghar A. Factors Influencing Transition to Shisheh (Methamphetamine) among Young People Who Use Drugs in Tehran: A Qualitative Study. Journal of psychoactive drugs. 2018;50(3):214–23. 10.1080/02791072.2018.1425808 29377788

[2] United Nations Office on Drugs and Crime, World Drug Report 2016 (United Nations publication, Sales No. E.16.XI.7).

[3] Goldstein RZ, Volkow ND. Dysfunction of the prefrontal cortex in addiction: neuroimaging findings and clinical implications. Nature reviews neuroscience. 2011;12(11):652 10.1038/nrn3119 22011681 PMC3462342

[4] Jiang G, Wen X, Qiu Y, Zhang R, Wang J, Li M, et al Disrupted topological organization in whole-brain functional networks of heroin-dependent individuals: a resting-state FMRI study. PLoS One. 2013;8(12):e82715 10.1371/journal.pone.0082715 24358220 PMC3866189

[5] Ma N, Liu Y, Li N, Wang C-X, Zhang H, Jiang X-F, et al Addiction related alteration in resting-state brain connectivity. Neuroimage. 2010;49(1):738–44. 10.1016/j.neuroimage.2009.08.037 19703568 PMC2764798

[6] Başar E, Schmiedt-Fehr C, Mathes B, Femir B, Emek-Savaş D, Tülay E, et al What does the broken brain say to the neuroscientist? Oscillations and connectivity in schizophrenia, Alzheimer's disease, and bipolar disorder. International Journal of Psychophysiology. 2016;103:135–48. 10.1016/j.ijpsycho.2015.02.004 25660302

[7] Sutherland MT, McHugh MJ, Pariyadath V, Stein EA. Resting state functional connectivity in addiction: lessons learned and a road ahead. Neuroimage. 2012;62(4):2281–95. 10.1016/j.neuroimage.2012.01.117 22326834 PMC3401637

[8] Mohan A, De Ridder D, Vanneste S. Graph theoretical analysis of brain connectivity in phantom sound perception. Scientific reports. 2016;6:19683 10.1038/srep19683 26830446 PMC4735645

[9] Wang Z, Suh J, Li Z, Li Y, Franklin T, O’Brien C, et al A hyper-connected but less efficient small-world network in the substance-dependent brain. Drug and alcohol dependence. 2015;152:102–8. 10.1016/j.drugalcdep.2015.04.015 25957794 PMC4458212

[10] Mahmoodi M, Abadi BM, Khajepur H, Harirchian MH, editors. A robust beamforming approach for early detection of readiness potential with application to brain-computer interface systems. 2017 39th Annual International Conference of the IEEE Engineering in Medicine and Biology Society (EMBC); 2017: IEEE. 10.1109/EMBC.2017.8037483 29060524

[11] Maryam Yasaminshirazi MA. Neuroimaging Findings in Methamphetamine Abusers. Addict Res Ther 2016.

[12] Newton TF, Cook IA, Kalechstein AD, Duran S, Monroy F, Ling W, et al Quantitative EEG abnormalities in recently abstinent methamphetamine dependent individuals. Clinical Neurophysiology. 2003;114(3):410–5. 10.1016/s1388-2457(02)00409-1 12705421

[13] Ahmadlou M, Ahmadi K, Rezazade M, Azad-Marzabadi E. Global organization of functional brain connectivity in methamphetamine abusers. Clinical neurophysiology. 2013;124(6):1122–31. 10.1016/j.clinph.2012.12.003 23332777

[14] Rubinov M, Sporns O. Complex network measures of brain connectivity: uses and interpretations. Neuroimage. 2010;52(3):1059–69. 10.1016/j.neuroimage.2009.10.003 19819337

[15] Sporns O, Honey CJ. Small worlds inside big brains. Proceedings of the National Academy of Sciences. 2006;103(51):19219–20. 10.1073/pnas.0609523103 PMC1748207 17159140

[16] Bassett DS, Meyer-Lindenberg A, Achard S, Duke T, Bullmore E. Adaptive reconfiguration of fractal small-world human brain functional networks. Proceedings of the National Academy of Sciences. 2006;103(51):19518–23. 10.1073/pnas.0606005103 PMC1838565 17159150

[17] Zhang J, Wang J, Wu Q, Kuang W, Huang X, He Y, et al Disrupted brain connectivity networks in drug-naive, first-episode major depressive disorder. Biological psychiatry. 2011;70(4):334–42. 10.1016/j.biopsych.2011.05.018 21791259

[18] Li C, Huang B, Zhang R, Ma Q, Yang W, Wang L, et al Impaired topological architecture of brain structural networks in idiopathic Parkinson’s disease: a DTI study. Brain imaging and behavior. 2017;11(1):113–28. 10.1007/s11682-015-9501-6 26815739

[19] Wang J, Zuo X, Dai Z, Xia M, Zhao Z, Zhao X, et al Disrupted functional brain connectome in individuals at risk for Alzheimer's disease. Biological psychiatry. 2013;73(5):472–81. 10.1016/j.biopsych.2012.03.026 22537793

[20] Ma S, Calhoun VD, Eichele T, Du W, Adalı T. Modulations of functional connectivity in the healthy and schizophrenia groups during task and rest. Neuroimage. 2012;62(3):1694–704. 10.1016/j.neuroimage.2012.05.048 22634855 PMC3408853

[21] Mohagheghian F, Makkiabadi B, Jalilvand H, Khajehpoor H, Samadzadehaghdam N, Eqlimi E, et al Computer-aided tinnitus detection based on brain network analysis of EEG functional connectivity. Journal of Biomedical Physics and Engineering. 2018. 10.31661/jbpe.v0i0.937 PMC6943854 32039100

[22] Yuan K, Qin W, Liu J, Guo Q, Dong M, Sun J, et al Altered small-world brain functional networks and duration of heroin use in male abstinent heroin-dependent individuals. Neuroscience Letters. 2010;477(1):37–42. 10.1016/j.neulet.2010.04.032 20417253

[23] Hsu T-W, Wu CW, Cheng Y-F, Chen H-L, Lu C-H, Cho K-H, et al Impaired small-world network efficiency and dynamic functional distribution in patients with cirrhosis. PLoS One. 2012;7(5):e35266 10.1371/journal.pone.0035266 22563460 PMC3341390

[24] Achard S, Bullmore E. Efficiency and cost of economical brain functional networks. PLoS computational biology. 2007;3(2):e17 10.1371/journal.pcbi.0030017 17274684 PMC1794324

[25] Meunier D, Achard S, Morcom A, Bullmore E. Age-related changes in modular organization of human brain functional networks. Neuroimage. 2009;44(3):715–23. 10.1016/j.neuroimage.2008.09.062 19027073

[26] Delorme A, Makeig S. EEGLAB: an open source toolbox for analysis of single-trial EEG dynamics including independent component analysis. Journal of neuroscience methods. 2004;134(1):9–21. 10.1016/j.jneumeth.2003.10.009 15102499

[27] Vinck M, Oostenveld R, Van Wingerden M, Battaglia F, Pennartz CM. An improved index of phase-synchronization for electrophysiological data in the presence of volume-conduction, noise and sample-size bias. Neuroimage. 2011;55(4):1548–65. 10.1016/j.neuroimage.2011.01.055 21276857

[28] González GF, Van der Molen M, Žarić G, Bonte M, Tijms J, Blomert L, et al Graph analysis of EEG resting state functional networks in dyslexic readers. Clinical Neurophysiology. 2016;127(9):3165–75. 10.1016/j.clinph.2016.06.023 27476025

[29] Hardmeier M, Hatz F, Bousleiman H, Schindler C, Stam CJ, Fuhr P. Reproducibility of functional connectivity and graph measures based on the phase lag index (PLI) and weighted phase lag index (wPLI) derived from high resolution EEG. PLoS One. 2014;9(10):e108648 10.1371/journal.pone.0108648 25286380 PMC4186758

[30] Xing M, Tadayonnejad R, MacNamara A, Ajilore O, DiGangi J, Phan KL, et al Resting-state theta band connectivity and graph analysis in generalized social anxiety disorder. NeuroImage: Clinical. 2017;13:24–32. 27920976 10.1016/j.nicl.2016.11.009 PMC5126152

[31] Ewald A, Aristei S, Nolte G, Rahman RA. Brain oscillations and functional connectivity during overt language production. Frontiers in psychology. 2012;3:166 10.3389/fpsyg.2012.00166 22701106 PMC3369188

[32] Haufe S, Nikulin VV, Müller K-R, Nolte G. A critical assessment of connectivity measures for EEG data: a simulation study. Neuroimage. 2013;64:120–33. 10.1016/j.neuroimage.2012.09.036 23006806

[33] Hjorth B. An on-line transformation of EEG scalp potentials into orthogonal source derivations. Electroencephalography and clinical neurophysiology. 1975;39(5):526–30. 10.1016/0013-4694(75)90056-5 52448

[34] Hu B, Dong Q, Hao Y, Zhao Q, Shen J, Zheng F. Effective brain network analysis with resting-state EEG data: a comparison between heroin abstinent and non-addicted subjects. Journal of neural engineering. 2017;14(4):046002 10.1088/1741-2552/aa6c6f 28397708

[35] Watts DJ, Strogatz SH. Collective dynamics of ‘small-world’networks. nature. 1998;393(6684):440 10.1038/30918 9623998

[36] Bullmore E, Sporns O. Complex brain networks: graph theoretical analysis of structural and functional systems. Nature Reviews Neuroscience. 2009;10(3):186 10.1038/nrn2575 19190637

[37] Beudel M, Tjepkema-Cloostermans MC, Boersma JH, van Putten MJ. Small-world characteristics of EEG patterns in post-anoxic encephalopathy. Frontiers in neurology. 2014;5:97 10.3389/fneur.2014.00097 24982649 PMC4058708

[38] Mohan A, De Ridder D, Vanneste S. Emerging hubs in phantom perception connectomics. NeuroImage: Clinical. 2016;11:181–94. 26955514 10.1016/j.nicl.2016.01.022 PMC4761655

[39] Pascual-Marqui RD. Standardized low-resolution brain electromagnetic tomography (sLORETA): technical details. Methods Find Exp Clin Pharmacol. 2002;24(Suppl D):5–12. 12575463

[40] Tzourio-Mazoyer N, Landeau B, Papathanassiou D, Crivello F, Etard O, Delcroix N, et al Automated anatomical labeling of activations in SPM using a macroscopic anatomical parcellation of the MNI MRI single-subject brain. Neuroimage. 2002;15(1):273–89. 10.1006/nimg.2001.0978 11771995

[41] Pascual-Marqui RD, Lehmann D, Koukkou M, Kochi K, Anderer P, Saletu B, et al Assessing interactions in the brain with exact low-resolution electromagnetic tomography. Philosophical transactions Series A, Mathematical, physical, and engineering sciences. 2011;369(1952):3768–84. 10.1098/rsta.2011.0081 21893527

[42] Imperatori C, Della Marca G, Brunetti R, Carbone GA, Massullo C, Valenti EM, et al Default Mode Network alterations in alexithymia: an EEG power spectra and connectivity study. Scientific reports. 2016;6:36653 10.1038/srep36653 27845326 PMC5109184

[43] Xia M, Wang J, He Y. BrainNet Viewer: a network visualization tool for human brain connectomics. PloS one. 2013;8(7):e68910 10.1371/journal.pone.0068910 23861951 PMC3701683

[44] Yun K, Park HK, Kwon DH, Kim YT, Cho SN, Cho HJ, et al Decreased cortical complexity in methamphetamine abusers. Psychiatry research. 2012;201(3):226–32. 10.1016/j.pscychresns.2011.07.009 22445216

[45] Khajehpour H, Mohagheghian F, Ekhtiari H, Makkiabadi B, Jafari AH, Eqlimi E, et al Computer-aided classifying and characterizing of methamphetamine use disorder using resting-state EEG. Cognitive Neurodynamics. 2019:1–12. 10.1007/s11571-018-9509-x 31741689 PMC6825232

[46] Mantini D, Vanduffel W. Emerging roles of the brain’s default network. The Neuroscientist. 2013;19(1):76–87. 10.1177/1073858412446202 22785104

[47] Engel AK, Fries P, Singer W. Dynamic predictions: oscillations and synchrony in top–down processing. Nature Reviews Neuroscience. 2001;2(10):704 10.1038/35094565 11584308

[48] Engel AK, König P, Kreiter AK, Schillen TB, Singer W. Temporal coding in the visual cortex: new vistas on integration in the nervous system. Trends Neurosci. 1992;15(6):218–26. 10.1016/0166-2236(92)90039-b 1378666

[49] Fries P. A mechanism for cognitive dynamics: neuronal communication through neuronal coherence. Trends in cognitive sciences. 2005;9(10):474–80. 10.1016/j.tics.2005.08.011 16150631

[50] Fries P. Neuronal gamma-band synchronization as a fundamental process in cortical computation. Annual review of neuroscience. 2009;32:209–24. 10.1146/annurev.neuro.051508.135603 19400723

[51] Jensen O, Kaiser J, Lachaux J-P. Human gamma-frequency oscillations associated with attention and memory. Trends in neurosciences. 2007;30(7):317–24. 10.1016/j.tins.2007.05.001 17499860

[52] Neuner I, Arrubla J, Werner CJ, Hitz K, Boers F, Kawohl W, et al The default mode network and EEG regional spectral power: a simultaneous fMRI-EEG study. PLoS One. 2014;9(2):e88214 10.1371/journal.pone.0088214 24505434 PMC3914938

[53] Burgess AP, Gruzelier JH. Short duration synchronization of human theta rhythm during recognition memory. Neuroreport. 1997;8(4):1039–42. 10.1097/00001756-199703030-00044 9141088

[54] Knyazev GG. Motivation, emotion, and their inhibitory control mirrored in brain oscillations. Neuroscience & Biobehavioral Reviews. 2007;31(3):377–95. 17145079 10.1016/j.neubiorev.2006.10.004

[55] Steriade M, McCormick DA, Sejnowski TJ. Thalamocortical oscillations in the sleeping and aroused brain. Science. 1993;262(5134):679–85. 10.1126/science.8235588 8235588

[56] Ronconi L, Oosterhof NN, Bonmassar C, Melcher D. Multiple oscillatory rhythms determine the temporal organization of perception. Proceedings of the National Academy of Sciences. 2017:201714522. 10.1073/pnas.1714522114 PMC5754799 29203678

[57] Engel AK, Fries P. Beta-band oscillations—signalling the status quo? Current opinion in neurobiology. 2010;20(2):156–65. 10.1016/j.conb.2010.02.015 20359884

[58] Csicsvari J, Jamieson B, Wise KD, Buzsáki G. Mechanisms of gamma oscillations in the hippocampus of the behaving rat. Neuron. 2003;37(2):311–22. 10.1016/s0896-6273(02)01169-8 12546825

[59] Alcaro A, Panksepp J. The SEEKING mind: primal neuro-affective substrates for appetitive incentive states and their pathological dynamics in addictions and depression. Neuroscience & Biobehavioral Reviews. 2011;35(9):1805–20. 21396397 10.1016/j.neubiorev.2011.03.002

[60] HajiHosseini A, Rodríguez-Fornells A, Marco-Pallarés J. The role of beta-gamma oscillations in unexpected rewards processing. Neuroimage. 2012;60(3):1678–85. 10.1016/j.neuroimage.2012.01.125 22330314

[61] Zilverstand A, Huang AS, Alia-Klein N, Goldstein RZ. Neuroimaging Impaired Response Inhibition and Salience Attribution in Human Drug Addiction: A Systematic Review. Neuron. 2018;98(5):886–903. 10.1016/j.neuron.2018.03.048 29879391 PMC5995133

[62] Goldstein RZ, Volkow ND. Drug addiction and its underlying neurobiological basis: neuroimaging evidence for the involvement of the frontal cortex. American Journal of Psychiatry. 2002;159(10):1642–52. 10.1176/appi.ajp.159.10.1642 12359667 PMC1201373

[63] Walsh ND, Phillips ML. Interacting outcome retrieval, anticipation, and feedback processes in the human brain. Cerebral Cortex. 2009;20(2):271–81. 10.1093/cercor/bhp098 19429861 PMC2803730

[64] Ishai A, Ungerleider LG, Martin A, Schouten JL, Haxby JV. Distributed representation of objects in the human ventral visual pathway. Proceedings of the National Academy of Sciences. 1999;96(16):9379–84. 10.1073/pnas.96.16.9379 PMC17791 10430951

[65] Herath P, Kinomura S, Roland PE. Visual recognition: evidence for two distinctive mechanisms from a PET study. Human brain mapping. 2001;12(2):110–9. 10.1002/1097-0193(200102)12:2<110::aid-hbm1008>3.0.co;2-0 11169875 PMC6871813

[66] Ray S, Hanson C, Hanson SJ, Bates ME. fMRI BOLD response in high-risk college students (part 1): during exposure to alcohol, marijuana, polydrug and emotional picture cues. Alcohol and alcoholism. 2010;45(5):437–43. 10.1093/alcalc/agq042 20729530 PMC2930251

[67] Tau GZ, Marsh R, Wang Z, Torres-Sanchez T, Graniello B, Hao X, et al Neural correlates of reward-based spatial learning in persons with cocaine dependence. Neuropsychopharmacology. 2014;39(3):545 10.1038/npp.2013.189 23917430 PMC3895231

[68] Chen Q, Zheng D, Cui S, Yan K-J, Fan C-x, Zhang G-f, et al Disrupted Resting-State Brain Functional Architecture in Amphetamine-Type Stimulant Abusers. Neuropsychiatry. 2018;8(1):249–60.

[69] BassettDS B. Humanbrainnetworksinhealthanddi ⋅ sease. CurrentOpinioninNeurology. 2009;22(4):340.

[70] Balconi M, Campanella S, Finocchiaro R. Web addiction in the brain: Cortical oscillations, autonomic activity, and behavioral measures. Journal of behavioral addictions. 2017;6(3):334–44. 10.1556/2006.6.2017.041 28718301 PMC5700716

[71] Hanlon CA, Dowdle LT, Naselaris T, Canterberry M, Cortese BM. Visual cortex activation to drug cues: a meta-analysis of functional neuroimaging papers in addiction and substance abuse literature. Drug and alcohol dependence. 2014;143:206–12. 10.1016/j.drugalcdep.2014.07.028 25155889 PMC4161649

[72] Florin E, Gross J, Pfeifer J, Fink GR, Timmermann L. The effect of filtering on Granger causality based multivariate causality measures. Neuroimage. 2010;50(2):577–88. 10.1016/j.neuroimage.2009.12.050 20026279

[73] Park SM, Lee JY, Kim YJ, Lee J-Y, Jung HY, Sohn BK, et al Neural connectivity in Internet gaming disorder and alcohol use disorder: a resting-state EEG coherence study. Scientific reports. 2017;7(1):1333 10.1038/s41598-017-01419-7 28465521 PMC5430990

[74] Barabási A-L, Gulbahce N, Loscalzo J. Network medicine: a network-based approach to human disease. Nature reviews genetics. 2011;12(1):56 10.1038/nrg2918 21164525 PMC3140052

[75] Zeng H, Su D, Wang P, Wang M, Vollstadt-Klein S, Chen Q, et al The Action Representation Elicited by Different Types of Drug-Related Cues in Heroin-Abstinent Individuals. Frontiers in behavioral neuroscience. 2018;12:123 10.3389/fnbeh.2018.00123 30013467 PMC6037213

[76] Lee JY, Park SM, Kim YJ, Kim DJ, Choi S-W, Kwon JS, et al Resting-state EEG activity related to impulsivity in gambling disorder. Journal of behavioral addictions. 2017;6(3):387–95. 10.1556/2006.6.2017.055 28856896 PMC5700729

[77] Jena SK. Examination stress and its effect on EEG. Int J Med Sci Pub Health. 2015;11(4):1493–7.

[78] Dluzen DE, Liu B. Gender differences in methamphetamine use and responses: a review. Gender medicine. 2008;5(1):24–35. 10.1016/s1550-8579(08)80005-8 18420163

[79] Berman S, O'Neill J, Fears S, Bartzokis G, London ED. Abuse of amphetamines and structural abnormalities in the brain. Annals of the New York Academy of Sciences. 2008;1141:195–220. 10.1196/annals.1441.031 18991959 PMC2769923

